# Advances in the Biotechnological Potential of Brazilian Marine Microalgae and Cyanobacteria

**DOI:** 10.3390/molecules25122908

**Published:** 2020-06-24

**Authors:** Deborah Terra de Oliveira, Ana Alice Farias da Costa, Fabíola Fernandes Costa, Geraldo Narciso da Rocha Filho, Luís Adriano Santos do Nascimento

**Affiliations:** 1 Institute of Biological Sciences, Graduation Program in Biotechnology, Universidade Federal do Pará, Augusto Corrêa Street, Guamá, Belém, PA 66075-110, Brazil; 2 Laboratory of Oils of the Amazon, Universidade Federal do Pará, Perimetral Avenue, Guamá, Belém, PA 66075-750, Brazil; analilice@hotmail.com (A.A.F.d.C.); geraldonrf@gmail.com (G.N.d.R.F.); 3 Institute of Exact and Natural Sciences, Graduation Program in Chemistry, Universidade Federal do Pará, Augusto Corrêa Street, Guamá, Belém, PA 66075-110, Brazil; 4 Campus of Salinópolis, Universidade Federal do Pará, Salinópolis, Pará, CEP 68721-000, Brazil; fabiolaffc@yahoo.com.br

**Keywords:** cyanobacteria, microalgae, marine, Brazil, biotechnology

## Abstract

Due the worldwide need to improve care for the environment and people, there is a great demand for the development of new renewable, sustainable, and less polluting technologies for food, health, and environmental industries. The marine environment is one of the main areas investigated in the search for alternatives to the raw materials currently used. Thereby, cyanobacteria and marine microalgae are microorganisms that are capable of producing a diverse range of metabolites useful for their cellular maintenance, but that also represent a great biotechnological potential. Due its great potential, they have an enormous appeal in the scientific research where, the biological activity of metabolites produced by these microorganisms, such as the antioxidant action of sterols are, some examples of biotechnological applications investigated around the world. Thereby, Brazil due to its extensive biodiversity, has high potential as a raw material supplier of marine waters, researching cyanobacteria and microalgae metabolites and their applications. Thus, this rapid review intends to present some important contributions and advances from Brazilian researchers, using the biomass of Brazilian cyanobacteria and marine microalgae, in order to illustrate the value of what has already been discovered and the enormous potential of what remains unexplored so far.

## 1. Introduction

Microalgae and cyanobacteria are a diverse group of microorganisms that can perform photosynthesis and can be found in different environments due to their capacity for adaptation. They have been widely studied for their storage of metabolites that exhibit biological activity and are the target of companies that produce pharmaceuticals, cosmetics, food, or fuels [1,2,3,4,5].

The search for these new resources largely involves the biotechnology and bioprospecting of microorganisms that are not toxic to the environment in which they live and that present advantages in their production. The researches commonly involve interest for metabolites with biological activity and, for a high-potential of biomass which are produced by these microorganisms, making them an alternative raw material to existing ones [1,3,6]. 

In the search for inputs, it is well known that the marine environment is abundant in organisms that produce substances with high biotechnological value; enable a wide variety of products and which can be used as possible solutions to diseases due to the discovery of new substances with biological activity. Therefore, there is a great demand for different species of marine cyanobacteria and microalgae which may have unknown substances or untapped actions with pharmacological potential [6,7,8,9].

Brazil is a country with great microbiological biodiversity, and it is a signatory to the Nagoya protocol [10], which regulates the guidelines for access and transfer of genetic resources in several countries and is an extremely important tool in controlling access to genetic heritage and preserving biodiversity. 

Due to its vast territorial expanse, Brazil generates a great diversity of biomes, including the marine environment, which are abundant sources of microorganisms, many of which are still unknown. Brazil so far it presents its own System to control access to genetic heritage National System for the Management of Genetic Heritage (SisGen) and applies these laws and decrees in the preservation of its biodiversity and to allow, in an assisted way, the creation of rules and terms for access to genetic information so that the genetic resources from its territory can be studied in partnership with other countries, generating products and services for society [11].

Substances that the marine cyanobacteria and microalgae produce and accumulate can be useful and exploited by the biotechnological market, like carbohydrates for ethanol production, hydrocarbons transformed in bio-oil, and lipids investigated for biofuel and aquaculture [12,13,14,15]. 

The pigments that they accumulate, like chlorophyll and the phycobiliproteins, are also an important focus in the research of animal food and supplements. Because of the role of these microorganisms in the production of metabolites that can be transformed, they are currently being modified and investigated [16,17,18].

This short review seeks to show what is currently being investigated regarding the potential that marine cyanobacteria and microalgae of the Brazilian region have as biotechnological resources.

## 2. Microalgae and Cyanobacteria as Metabolite Producers

**Figure 1:**
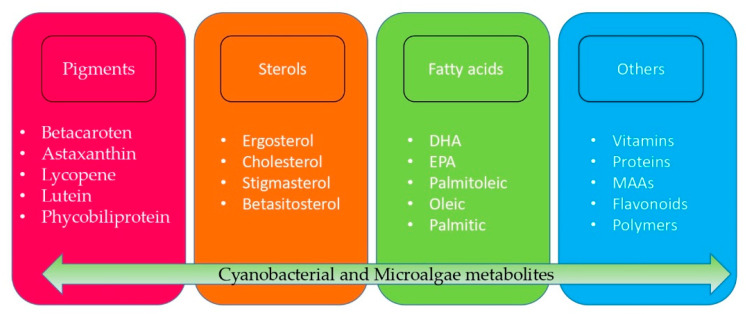
Metabolites produced by cyanobacteria and marine microalgae. DHA: Docosahexaenoic acid; EPA: Eicosapentaenoic acid; MAAs: Mycosporine-like amino acids [1,3,7,15,16].

Metabolites produced by cyanobacteria and microalgae have been studied in recent decades as biotechnological products with high commercial value, having been observed in their first use as food by the Chinese as a food product, and nowadays, they are also a source of fatty acids, pigments, carotenoids, and proteins that are widely researched for cosmetic products, like supplements for human and animals, some metabolites can be a precursor for drugs against bacteria, fungi or used in treatment for plants (Figure 1) [15,16].

**Figure 2:**
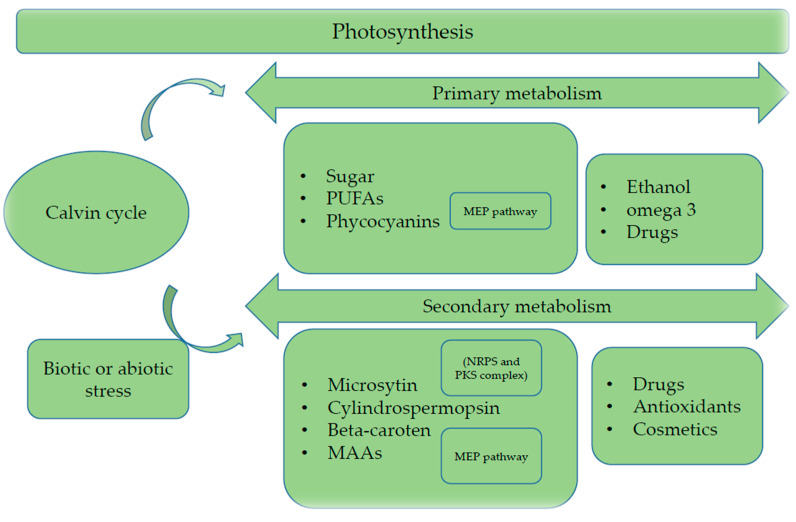
Primary and secondary metabolism of microalgae and cyanobacteria. They apply the MEP pathway to generate PUFAs and proteins in the primary metabolism and, when suffer some biotic or abiotic stress can produce beta-carotenes, microcystin and other substances [22,23,24].

The primary metabolism of microalgae and cyanobacteria are responsible for providing substances that will be the raw material for the production of biofuels such as ethanol and biodiesel, producing fatty acids that are the basis for the production of omega and proteins with biological activity (Figure 2). As well as proteins that are strongly studied as antioxidant agents or their antibacterial and antifungal properties, being a great target for the production of medicines, whether for agriculture in the fight against pests or in the treatment of human diseases of animals [19,20,21]. 

Because they are found in different environments, one of the cellular structures responsible for the environmental adaptation of microalgae and cyanobacteria to the environment is the phycobilisome, in which proteins called phycobiliproteins, carotenoids and chlorophylls, which are involved in light capture and cell photoprotection, are present and are seen as potential precursors to drugs that fight bacteria, viruses and cancer cells [25,26,27,28].

In addition to participating in cell photoprotection, when stimulated by some biotic or abiotic factor, the secondary metabolism of microalgae and cyanobacteria is aroused for the production of some metabolites that may have biological activities of great biotechnological importance, such as sterols, toxins and some amino acids that have the ability to activate viral capsule structures, preventing their cell multiplication, thereby marking an antiviral agent (Figure 2) [7,19,29].

Borowitzka [30], made a survey about proteins, carotenoids, sterols and fatty acids, highlighting the importance of the production of beta-carotene by microalgae in US and Australian companies, as a natural carotenoid that competes with that produced synthetically. Further work has been conducted by Manning and Nobles [31], who evaluated cyanotoxins like microcystins and cylindrospermopsin and their toxicity in the waters of effluents and the danger they pose. 

Many marine microorganisms represent great potential for the production of metabolites with biological activities that are still unknown, and research on these raw materials is still scarce; however, work in this field has increased due to the unlimited source provided by marine waters. 

Recently, Saini et al. [32] also demonstrated cyanobacteria as major producers of pigments that function in cellular protection and microalgae that can be used as natural pigments such as phycobiliproteins and carotenoids, replacing synthetic dyes, which have antioxidant activity and can be used in the composition of food and pharmaceutical and cosmetic products. 

In Table 1, the work described by Li et al. [33] showed that phycocyanin-c (C-PC) from *Spirulina platensis* obtained from the Laboratory of Algal Biotechnology in the Ocean University of China, may have antitumor and anticancer actions—inducing the gene production stimulates the cell apoptosis and also induces the downregulation of anti-apoptotic gene expression, resulting in the death of tumoral cells like HeLa that were investigated. These results reveal that C-PC may be a candidate for a natural agent, as a substitute for chemotherapy agents or, likewise, in current therapy combinations for diseases. 

The secondary metabolism of cyanobacteria and microalgae is responsible for producing substances that arise only in the presence of environmental stress or with genetic modifications, such as the insertion of genes like carotenoids production by light stress and the tyrosine ammonia lyase gene to p-coumaric acid and that can be beneficial to human health [34,35].

Taori et al. [36], which evaluated the production of largazole, an anti-cancer agent, synthesized by secondary cyanobacteria metabolism of *Symploca* sp. that was collected from Key Largo (Florida Keys, USA) that has antiproliferative activity, inhibiting growth of a breast cancer model cell (GI_50_ 7.7 nM) and can induce cytotoxicity with higher concentrations (LC_50_ 117 nM). 

**Figure 3:**
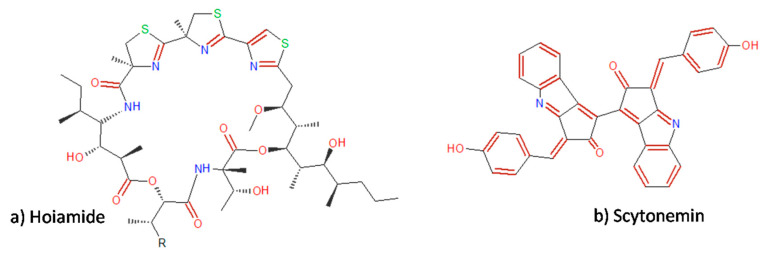
(**a**) Hoiamide, a peptide produced by cyanobacteria with antifungal, cytotoxic and neurotoxic properties [37]. (**b**) Scytonemin structure [41].

The studies of Li et al. [37], evaluated too some methods of hoiamide synthesis (Figure 3a), another antitumoral substance made by cyanobacteria *Moorea producens* and *Phormidium gracile* initially isolated from Papua New Guinea that has cytotoxity ability. Its biological activity, as well as other substances with biological activity, is strongly related to the chemical structure it has, its chiral centers and the chemical reactions that are carried out in the presence of double bonds and atoms arranged in the structure showing its potential for the biotechnology and pharmaceutical markets [38,39,40].

In the continued search for new natural products, scytonemin (Figure 3b) is a cyanobacterial lipid-soluble substance that has high cell activity protection against UV radiation but is only found in cyanobacteria. Rastogi et al. [1] investigated and identified scytonemin produced by *Lyngbya* sp. and obtained of a bark sample from *Albizia saman* (Jacq) Merr from Thailand, and suggested a possible application of different forms of scytonemin in the composition of cosmetic products with UV protection due to its antioxidant activity reaching 57% of the activity (Table 1).

Matsui et al. [42] evaluated other pigments produced by *Nostoc commune* that were collected from the Tsubata Shinrin Kohen (Ishikawa, Japan), including mycosporine-like amino acids (MAAs), which are water-soluble pigments that act against ultraviolet (UV) radiation and photo-oxidation of combat cells. The 27% contribution to the total radical-scavenging activity of the extract showed that, when compared with the current agents of antioxidant activity like Trolox, MAAs are great candidates for the composition of cosmetic products with cellular protection activity. They can be found in various organisms, from bacteria to lichens, fungi, and animals. 

All of these cyanobacterial pigments showed potential for use in biotechnology and reached a global demand from consumers in relation to the use of natural products that are not toxic or environmental pollutants. 

Accordingly, Zhu et al. [43] the identified carotenoids, zeaxanthin and echinenone, in mutant cyanobacteria *Synechococcus* 7002, act in the cyanobacterial photosynthesis process like a protective agent from cellular photo-oxidation. They showed that the carotenoids are able to accumulate high levels of ROS (reactive oxygen species) and RNS (reactive nitrogen species). 

Some results show that after light exposure, the mutants produced large quantities of the enzyme genes that participate in carotenogenesis, indicating greater production of carotenoids and higher protection of the cell against oxidative stress and, highlighting that the carotenoid has a huge biotechnological role to be developed.

**Figure 4:**
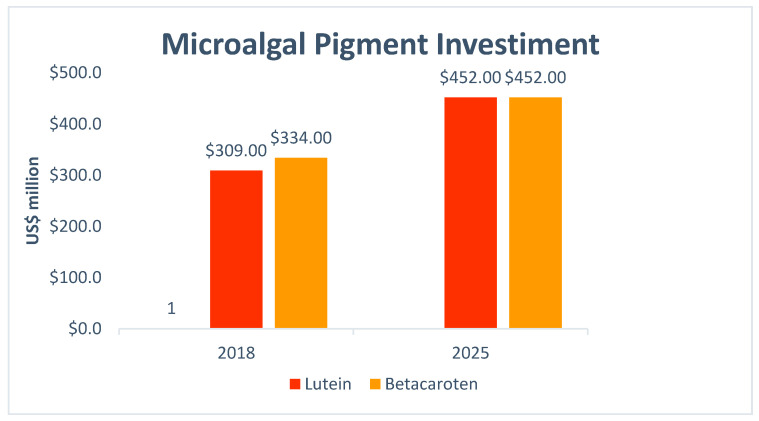
Total World investments (2018–2025) on microalgal pigments [45].

Figure 4 shows the worldwide investment in the production of pigments from microalgae from 2018 until 2025, highlighting the use of β-carotenes and lutein, which are pigments widely used as dyes and in the composition of animal and human foods [44,45].

In addition to pigments of cyanobacteria and microalgae, these microorganisms can produce other substances that are part of their secondary metabolism and which may be of interest to the biotechnology industry (Table 1). 

Fagundes et al. [46], evaluated the production of sterols with antioxidant activity present in the cyanobacterium *Phormidium autumnale*, that were first isolated from the Cuatro Cienegas desert (Mexico) and, found stigmasterol (455.3 µg g^−1^) and also β-sitosterol (279.0 µg g^−1^), which are known by their high antioxidant activity and are produced by using different external carbon sources. 

The work by Badr et al. [47] investigated either the phenolic compounds produced by cyanobacteria *S. aphanizomenoides* isolated from irrigated agriculture cannels located at Kafr Elsheikh Governorate (Egypt) like flavonoids and carotenoids that showed an extensive therapeutic effect against oxidative stress damage with 58.74% of scavenging DPPH activity. 

As shown in Table 1, the work of Santos-Merino [48] analyzed the role of enzymes in the higher production of fatty acids from primary metabolism by genetically modified cyanobacteria *Synechococcus elongatus* PCC7942 that produce omega-3 for supplements. 

These cyanobacterial metabolites for presenting interesting values of biological activity, have greater economic value, and exemplify how the food, cosmetics and pharmaceutical industries can be increasingly favored by studies that reveal the great potential of this microrganisms.

In Brazil, the research demand for microorganisms as raw material is mostly related to bacteria, fungi and yeast. Investigations into microalgae and cyanobacteria are quite recent and address a range of interests, ranging from looking for biologically active substances to their pharmaceutical, nutraceutical and energy market applications [7,8,49,50]. However, a survey conducted in this work on intellectual property protection in Brazil revealed that, the use of marine cyanobacterial biomass as raw material is currently largely focused on the food market, particularly composing and, being the basis of some food supplements, according to data of the national patent registration bank in Brazil, certified by the National Institute of Industrial Property (INPI).

Most protected products and processes involve food composition because breaking down cyanobacterial cells is still an expensive procedure and needs further research and studies involving this stage. The *Spirulina* genus is hardly studied in Brazil, with most patents worked with it due to the fact that its species are well known and widely studied worldwide. Other species like *Nostoc* and *Synechococcus* are often chosen for studies because they are almost always found when collections are carried out and also because their genomes have already been identified worldwide, which improves understanding in the research carried out by groups in Brazil [9,54]. 

In order to explain the great potential contained in these microorganisms, the next chapter reports on the scientific works involving cyanobacteria and microalgae as sources of metabolites of biotechnological interest developed by Brazilian researchers, through their most relevant results.

## 3. Cyanobacteria and Microalgae at Brazil 

### 3.1. Energy

In Brazil, soy and sugarcane are the most commonly used raw materials for biofuel production; however, there are drawbacks involved in the use of these vegetables for biofuel production, such as competition with the food market, the use of large planting areas, depletion of the soil in which the plants are and the time they need to grow. 

In this sense, the use of other renewable sources such as cyanobacteria and microalgae emerges as an alternative for the production of biofuels, as these bacteria have great potential for production of oils, easy genetic modification due to their uncomplicated genome, and grow in smaller spaces and thus require fewer nutrients than existing organisms used for oil production [55,56].

The production of biofuels using cyanobacteria and microalgae as a raw material involves varying the conditions under which they grow, and may use statistical models so that it is possible to optimize this production and ensure that microalgae and cyanobacteria can improve the scenario of the third generation of biofuels in Brazil.

Thus, Pereira et al. [57] used a statistical model to evaluate the best conditions for the production of fatty acid biomass from a *Chlorella minutissima* lineage in a bubble column photobioreactor. In this study, it was possible to reach the production of cellular biomass and fatty acids, being 57.1% palmitic acid (C16:0) and, 26.3% stearic acid (C18:1), adding 83.4% of the total fatty acids produced by the strain which are equal or superior to other microalgae already researched for this purpose. 

These results confirm that some studies about different conditions for cyanobacteria and microalgae development are essential to reach advantages and yield and to affirm the potential that this strain represents for the production of a third-generation biodiesel for the Brazilian market (Table 2). 

Similarly, Amaral et al. [58] described a study with strains of *Chlorella* sp. that a statistical model was also used to evaluate the best conditions of NaNO_3_ as a source of nitrogen and CO_2_ to increase the productivity of biomass with a high lipid content. A biodiesel produced by this biomass was evaluated and showed good parameters of fatty acid composition with large production of palmitic and oleic fatty acids (20.7% and 27.2, respectively) reaching a rate of 78.4% conversion of fatty acids into biodiesel by the transesterification method. 

Assessing the production of oil as triglycerides (TG) in strains of *Isochrysis galbana* and *Phaeodactylum tricornutum*, Kurpan Nogueira et al. [59] varied temperature and light in the cultures of the strains and observed a greater accumulation of TG, which can be transformed into biodiesel. When the microorganisms were exposed to high rates of light and low temperature, 300 µmol photons m^−2^ s^−1^ and, 20 °C to *P. tricornutum* and, 400 µmol photons m^−2^ s^−1^ and, 30 °C to *I. galbana*, reaching an increase of 39% and 70% respectively in oil production in biomass.

These works show that research in Brazil are very focused on optimizing the production of microbial biomass so that it is possible to reach a model that becomes competitive with the global market for the third generation of biofuels.

Nascimento et al. [60] carried out a study to compare the production of oil of ten marine microalgae and oleaginous vegetables, realizing that it is possible to achieve, from the *Chlorella* microalgae evaluated, a high-quality oil that guarantees the production of biodiesel with excellent qualities. 

They also highlighted the values of CN, a parameter of great importance for a biodiesel, varying from 48 to 65, and observed an excellent mixture of SFA and MUFAs, which attests to the high stability of the fuel. Despite the great presence of PUFAs in microalgae and cyanobacterial oils, strains with this profile are discarded for the synthesis of biodiesel but promise other biotechnological applications in the composition of animal feed or human food. 

These types of studies demonstrate the potential that exists in the use of microalgae and cyanobacteria biomass for energy production in Brazil, also indicating a resource that improves the process, adding value to the waste, decreasing the use of water and reagents, and generating products such as biofuels which are of high relevance to industry. 

Silva et al. [61] analyzed twelve different types of marine cyanobacteria to obtain their cellular composition for saponificable components. To *Synechococcus* sp., *Amphora* sp., *Planktolyngbya limnetica* and, *Buiddulphia* sp. the potential for biodiesel production and other uses, revealing that some of them are capable of producing up to 36.3% of dry biomass to be used as feedstock. While other lineages are, rich in inorganic elements that are not a good source for biofuels but are a good choice for fertilizer or animal food. 

Similarly, Calixto et al. [56], carried out a survey on the lipid composition of twelve marine cyanobacterial and microalgae strains from Northeastern Brazil. In particular the *Synechocystis* sp. strains, which have been shown to produce a great quantity of lipids (54.2 mg/L.d) composed of generally saturated and monosaturated fatty acids are, ideal for the production of a good quality biodiesel with stability and fluidity. 

In order to direct the fatty acid composition to be favorable for biodiesel production, Baumgartner et al. [62], conduct a search about parameters that can be modified in a synthesis of biodiesel from cyanobacterial culture. They varied parameters like the solvent for fatty acid transformation and, the time and temperature of reaction of the oils extracted of the cultivation of *Spirulina platensis* and its conversion into good biodiesel, obtaining excellent results from 742 g·mol^−1^ of average molecular mass of the oil. 

Currently, ethanol production in Brazil is carried out with sugarcane as the main raw material, but it is known that the amount of waste generated by this production leads to the use of microorganisms as substitutes for the raw material, which is of great importance for the national energy market [63]. 

Cyanobacteria, during photosynthesis, capture CO_2_ from the environment, lowering its concentration in the atmosphere, and produce carbohydrates, which can be used in bioethanol synthesis. In this way, some studies have been conducted in Brazil, such as the work of Klein et al. [64] who made an review about the attempt to insert the microalgae biomass cultivation into a large-scale production, considering the biorefinery process take the ethanol production with, carbon sources and other nutrients reutilized for them.

In which different parameters for microalgae biomass insertion were studied as well as how the difficulties encountered in using this biomass for energy production could be overcome [65,66].

Also worth mentioning are two works developed by Braga et al. [67,68], who considered different concentrations of nitrogen supply for the cultivation of *Spirulina* sp. and different carbon sources, aiming to increase carbohydrate production for its fermentation for bioethanol production. With different conditions, it was possible to achieve the production of 49.3% (*w*/*w*) of carbohydrates in the *Spirulina* sp. biomass under cultivation using carbon dioxide (CO_2_) as a carbon source. When fly ash and CO_2_ were used as carbon sources, they reached up to 63.3% of carbohydrates, also ensuring that bioethanol production using marine cyanobacterial biomass with CO_2_ assimilation can contribute to the reduction of environmental problems. 

Rempel et al. [69], with the intention of studying *Spirulina platensis* biomass as a source of renewable fuels, found that it was possible to achieve 83% fermentation of carbohydrates obtained from biomass for the production of bioethanol. Using the residual biomass from this production, biomethane was generated with a great energy potential, around 13945 kJ·kg^−1^, confirming the use of cyanobacteria biomass as a good source for the production of biofuels.

Among these studies carried out in Brazil, the use of wastewater as well as lignocellulosic material as nutritional supplementation has emerged as an alternative in the cheaper production of biofuels. In the work of Deprá et al. [70] the authors dealt with the concept of microalgal biomass biorefinery, which can give rise to several biofuels, including bioethanol, but also dealt with the use of waste material as a source of nutrients for the production of microorganism biomass. 

Some research in Brazil has also involved the use of marine microalgae biomass for energy production, as in the work of Santos et al. [71], in which cellular pretreatment parameters for methane production from *Isochrysis galbana* biomass were evaluated as reaching a 71.5% increase in methane yield with the biomass that pass for an acid pretreatment. This strain is known worldwide for providing raw material for carotenoid production due to its high rate of fucoxanthin production. Therefore, in order to obtain an energy-efficient industrial process, the biorefinery technique has been studied in this strain, seeking ways to get more produce from a crop.

However, the biomass of marine cyanobacteria, when compared to microalgae, has been explored less in research in Brazil, even though they are fewer complex organisms that require the same cultivation conditions, and the desired metabolites they produce are more accessible. 

Evaluating the cultivation of *Spirulina maxima* because of its great protein content, Simão et al. [72] carried out research on the hydrocarbon production of this strain and, its use in the production of bio-oil, achieving 51.52 kJ·mol^−1^ of energy activation from the biomass rich in carbohydrates and, 288.55 kJ·mol^−1^ from the biomass that had more protein in its composition. 

The technique used by the researchers, pyrolysis, is widely applied in lignocellulosic raw materials for the same purpose. However, its application in cyanobacteria biomass and the study of the reaction parameters performed in this work applying the pyrolysis were novel and proved to be efficient methods of obtaining aromatic hydrocarbons, which may be used in the composition of various products derived from bio-oil [73,74].

Thus, it is clear that, in Brazil, research with alternative sources for the production of biofuels aids in the removal of tailings from the environment, giving them utility, in addition to the possibility of the synthesis of biocompounds from marine cyanobacteria and microalgae that have high value to the chemical market as volatile organic compounds and biofuels.

### 3.2. Drugs

Cyanobacteria are microorganisms known to produce substances that can be of high economic value for medicine research. These substances are usually non-ribosomal peptide-synthesized, using enzymes known as non-ribosomal peptide synthetase (NRPS) as well as polyketide synthase I (PKS). Like scytovirin and microvirin, these substances are strong candidates for drug production and therapies because they have different targets of action, interacting with viruses, protozoa and bacteria (Figure 2) [75,76,77].

Along with these substances, cyanobacteria are known to synthesize a variety of metabolites with valuable biotechnological applications, such as their isoprenoids, pigments and antiproliferative substances that are of great importance for the global pharmaceutical market and, they are currently playing a relevant role in blue biotechnology as alternatives to be investigated worldwide [78,79,80,81]. 

Thinking about logic, Carmichael and Li [82], observed cyanobacteria that live in salt water have a higher occurrence in the production of toxins than those found in other environments, emphasizing that salinity can have a great influence on the production of the toxin like microcystins that can have biological activity against some microrganisms. In this sense, they are a great target in the investigation of new substances with pharmaceutical biotechnological potential.

In Brazil, a country where the occurrence of mosquito-borne viral diseases has increased every year, the Ministry of Health, only in 2019, warned the population about the increase of dengue cases, which had risen by about 282% in April this year compared to the same period in 2018 [83]. With the high incidence of diseases such as dengue, Zika, Chikungunya and yellow fever, research involving substances that can combat both the vector and the causative agent of these diseases has grown in this country [84,85,86,87,88].

With that situation, microorganisms producing secondary metabolites that show some action against the causes of these diseases represent targets of several studies. In this context, cyanobacteria are the target of research because they can produce peptides, flavonoids, and terpenoids, among other substances, that have insecticidal or larvicidal biological activity. 

**Figure 5:**
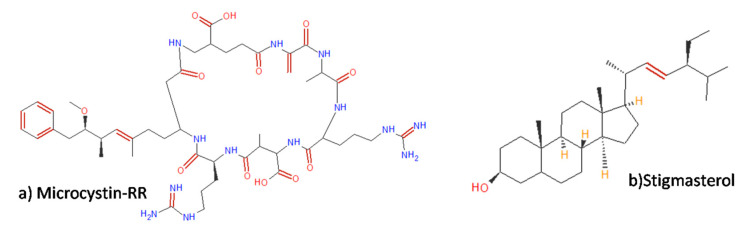
(**a**) Microcystin-RR, larvicidal substance [89]. (**b**) Stigmasterol isolated from cyanobacterium *Phormidium autumnale* that exhibits anti-inflammatory biological activity [93].

Geris et al. [89] conducted a survey on bioactive natural compounds that fight the multiplication of the mosquito *Aedes aegypti*, as well as the vector of some of these diseases, and revealed that the cyanobacterium *Microcystis aeruginosa* produces a peptide with larvicidal activity capable of fighting the mosquito. The peptide called microcystin-RR, illustrated in Figure 5a, has action in the larval stage of the mosquito and can act as a larvicide, a preventive of the diseases transmitted by *Aedes aegypti*.

Microcystins are cyanotoxins formed by cyclic peptides that are toxic to animals and humans. They are known for their primary action in liver cells, leading to necrosis. However, some researchers have already observed their action in other human organs, so based on research on its toxicity, the World Health Organization determined a limit for the presence of cyanobacteria that can produce cyanotoxins in water to ensure the health of aquatic animals as well as humans. In such cases, the toxicity of the substance should always be evaluated to cause no greater harm and can be tested in combating diseases [90,91,92].

Ramos et al. [94] evaluated a variant of microcystin that showed antimicrobial activity against mycobacteria. Some strains of *Mycobacterium tuberculosis* are known to cause tuberculosis in humans. In this sense, extracts of *Microcystis aeruginosa* containing the microcystis toxin were tested and, antimicrobial activity against *M. tuberculosis* with a minimum inhibitory concentration (MIC) of 1.93 µM was found with no cytotoxicity in these concentrations, showing this microcystin to be a promising candidate for the development of new therapies and drugs for the treatment of tuberculosis.

According to Scaglioni et al. [95], the search for metabolites to combat the *Fusarium* fungus, a pathogen commonly found in cereal crops, led to the search for phenolic compounds in *Spirulina* sp. and *Nannochloropsis* sp. This study reported a higher antifungal activity index and, a report on synthetic antifungals reaching about 93% of the activity for the extraction of *Spirulina* sp. and, 76% for *Nannohloropsis* sp., demonstrating the high potential represented by microalgae for the production of products with antifungal activity and for the agricultural industry.

Fagundes et al. [46] studied the secondary metabolites produced by the cyanobacterium *Phormidium autumnale* using different carbon sources, including wastewater, in an attempt to observe and quantify the production of substances with biological activity produced by cyanobacteria. In this search for metabolites, several steroids were identified, some of which had the ability to lower the cholesterol level in human blood and, thus are being marketed as drugs. Some steroids were produced in large quantities by cyanobacteria when wastewater was used as a carbon source; stigmasterol (Figure 5b), cholesterol, squalene, and β-sitosterol had the largest sterol profiles and have been reported as favorable antioxidant and antitumor substances [93].

Silva-Stenico et al. [96] conducted a survey on the production of metabolites with the biological activity of 411 cyanobacteria and microalgae, including marine species, and demonstrated that they produce substances with antiproliferative activities against cancer cells, such demonstrating 49% inhibition of Gram-negative pathogenic cells and, 35% inhibition of Gram-positive pathogenic cells, showing a huge potential in the biotechnological and pharmaceutical industry.

In the same way, Barboza et al. [54] evaluated the capacity of different marine cyanobacteria collected in waters and corals of Northeastern Brazil and found that *Synechocystis aquatilis*, *Synechococcus* spp. and *Romeria gracilis* show antimicrobial activity against bacteria *Staphylococcus aureus* and *Pseudomonas aeruginosa*, known for their pathogen activity, and evaluated the inhibition zone formed in the methanolic and ethanolic extracts. 

These kinds of studies revealed the great potential that is still unexplored in marine cyanobacteria off the Brazilian coast, which is surrounded by oceans, with salty waters that have a multitude of microorganisms to be explored and transformed into Brazilian biotechnological raw material.

### 3.3. Nutraceuticals

The demand for food and supplements that address the appearance of diseases in people related to poor nutrition in vitamins, omega-3 and antioxidant agents that are not produced by human metabolism is becoming increasingly common. In order to mitigate this problem, the consumption of natural food supplements has been effectively sought after by the population and nutraceuticals have arisen in response to this search, attenuating food shortages and avoiding the use of synthetic products [97]. 

In this search, microalgae and cyanobacteria have played an indispensable role in obtaining substances that make up these nutraceuticals. The microorganisms are sources of a wide variety of metabolites, such as pigments formed by carotenoids and proteins that can have antioxidant activities and are enormously important in cosmetics and food markets [98,99]. 

They also produce phenolic compounds with antifungal and bactericidal activities that have pharmacological potential as well as metabolites that contribute to a healthy diet, such as polyunsaturated fatty acids (PUFAs), with action in the control of high-density lipoprotein- HDL and low-density lipoprotein- LDL, which reduce the production of triglycerides. These compounds can also be used in anti-inflammatory drugs [100,101].

The practice of using microalgae as food has occurred since ancient times, especially in Asia and North America, and extends to the present day. With each decade, they have become promising candidates for protein supply, particularly in *Chlorella* and *Arthospira* pigment production and with a focus on β-carotene and astaxanthin in the cultivation of the *Dunaliella* and *Haematococcus* for several bioproducts such as human food additive and animal feed. 

In the early 1990s, some studies began on the production of polyunsaturated fatty acids with a focus on docosahexaenoic acid (DHA) and eicosapentaenoic acid (EPA) for use in aquaculture. These substances are used in the production of foods composed of various substances, such as vitamins, mineral salts, pigments, lipids, and fatty acids [100].

In Brazil, Gomes et al. [102] analyzed the advantages that the addition of the biomass of the marine cyanobacterium *Spirulina platensis*, rich in polyunsaturated acids, could add to the diet of red tilapias, which are fish of great economic potential in aquaculture.

With the presence of cyanobacteria in the feed, it was possible to verify that the fish obtained greater growth, weight gain and, consequently, a higher survival rate, benefits attributed to the presence of PUFAs from the biomass of *Spirulina platensis*, known for its ability to increase fish immunity among other benefits. 

In addition to these advantages, the color of animals fed with cyanobacteria also changed, with a stronger yellow in animals with dry biomass of cyanobacteria added to their diet, which is also an advantage in the food market. This change is attributed to the consumption of carotenoids also present in the biomass of cyanobacteria, thus reaffirming the advantages that the use of marine cyanobacteria in aquaculture can bring.

Magnotti et al. [103] designed an integrated multitrophic crop using wastewater from shrimp farming; *Litopenaeus vannamei* to grow marine microalgae; *Tetraselmis chuii*, *Nannochloropsis oculata*, and *Chaetoceros muelleri*, which supplemented crustacean crops; and *Artemia franciscana*, which was used as shrimp food. 

The greatest growth was observed in *A. franciscana* fed with *T. chuii*, which in the multitrophic system was the most advantageous, producing 0.83 g of *A. franciscana* biomass, saving the use of water and reducing the quantity of nutrients purchased. 

In this study, it is possible to observe the advantages brought about by the use of biomass of cyanobacteria and marine microalgae inserted in a production system that is already consolidated in Brazil in order to benefit the production in Brazilian aquaculture.

In Brazil, the work of Brasil and collaborators [104] evaluated that the microalgal lipid content can range from 1% to 40% of the dry weight and, under certain cultivation conditions, can reach up to 85% of lipids. It is possible to observe that these microalgae are attractive to researchers and industry due to their great capacity for generating polyunsaturated omega-3 and omega-6 long chain acids and, products with high benefit, making them promising raw materials for the production of food supplements. 

In the study conducted by Morais et al. [105], the conditions of cultivation of different cyanobacteria and marine microalgae were evaluated to be used as a lipid-rich food source. As well as the work of Campos et al. [98], in which it was evaluated that the lipid composition of cyanobacteria can be modulated according to the desired cultivation parameters and, conditions and compose dietary supplements rich in essential fatty acids, not produced by human metabolism and requiring supplementation like omega. 

In this sense, Feller et al. [106] also evaluated the antioxidant activity performed by strains of *Phaeodactylum tricornutum*, *Nannochloropsis oculata* and *Porphyridium cruentum*, which presented high rates of cell protection activity. They evaluated different techniques to extract carotenoids, PUFAs, and lipids from the lineages and their relation with scavenger activity. 

The supercritical CO_2_ (SC-CO_2_) technique was the best methodology to obtain carotenoids from biomass extracts, but the subcritical n-butane extraction showed the maximal scavenger activity, with 44.63% in *P. cruentrum* extract. These analyses contribute to a better understanding of the potential use of the metabolites and their level of performance.

Jaeschke et al. [99] showed that the main fatty acid found in *Heterochlorella luteoviridis* was palmitic acid, representing 35% of the total fatty acid content. Among all the fatty acids identified, 32% linoleic acid, 10% α-linoleic acid, 7% stearic acid and 6% oleic acid were found. In this work, was an interesting method of carotenoid and lipid extraction of the microalgae that showed high yield extraction and uses a less harmful and non-toxic solvent.

Sassi et al. [107] evaluated some freshwater and marine microalgae species and, their production of metabolites of biotechnological interest such as carbohydrates, pigments and lipids. They could point to the marine lineage *Amphinidiam carterae* as a strong producer of carbohydrates, producing about 29.31 g·100 g^−1^, it produces approximately 31.60 g·100 g^−1^ of proteins. As well as in the presence of environmental stimuli, is capable of producing large amounts of lipids and carotenoids, reaching 29.78 g·100 g^−1^ and 17.64 mg·100 g^−1^, respectively.

Metabolites like carotenoids have great commercial value in nutraceutical products, as they are pigments of great commercial interest and function as photoprotectors; each species of microalgae can contain between five and ten types of a universe of approximately 60 different carotenoids present in your cells. 

Several species can accumulate a large concentration of β-carotene, astaxanthin and cantaxanthin, which have a wide range of applications as natural dyes and as antioxidants. Ariede et al. [108] showed the use of algae in the cosmetics market and, cites the participation of cyanobacteria and microalgae like *Isochrisis* in the production of creams with protective action against UV radiation, *Spirulina* for anti-aging skin creams and, *Chlorella* shampoos, and emulsions with metabolites derived from marine cyanobacteria, confirming all of the potential of these microorganisms. 

The growing industrial interest in these natural pigments can be explained by the ability attributed to them to prevent degenerative diseases, fighting free radicals and functioning as anticancer agents and immune system stimulators. Compared to synthetic dyes, the natural pigments have some advantages because they are more resistant to the presence of ascorbic acid, to the heat and freezing processes, and present efficiency even when applied in foods in small quantities [109].

Table 3 shows some research conducted in Brazil focusing on the production of metabolites produced by cyanobacteria and marine microalgae. Derner et al. [109] evaluated a diversity of functions attributed to microalgae biomass and, described that in Brazil, the focus of research and companies has been the production of natural dyes for food.

Nunes and collaborators [110] showed that astaxanthin content was changed depending on the stimulus given to *H. pluvialis* (which was 4.2 times higher), and these products are found in the form of powder concentrates, lyophilized or dehydrated biomass, as well as extracts in vegetable oil and pigments that are very often applied in the food market [111].

Neto et al. [112] evaluated ways to cheapen the cultivation of *Nannochloropsis oculata* using low-cost culture media, composed of commercial fertilizers and also effluents from aquaculture. The production of chlorophyll-a was observed as the main pigment produced, but some carotenoids with strong commercial appeal such as zeaxanthin, violaxanthin, and β-carotene were also produced, ensuring a crop with less expenditure on nutrients for microalgae.

Currently, there is considerable effort in research and development to produce food products from microalgae. However, the most significant and critical barrier to the implementation in the market of commercially viable algae production remains the high cost of production, especially in relation to the value of cultivation and processing. In addition, in view of low process productivity, the microalgae industry will be forced to focus or rely on a multi-product biorefinery approach [114].

## 4. Conclusions and Prospects of Brazilian Biotechnology

This review elucidated the research conducted in Brazil by Brazilian researchers who deal with the vast biodiversity present in the Brazilian marine environment and that represent a huge potential for the biotechnological market in the country.

Considering that cyanobacteria and marine microalgae represent a large part of the marine environment and mainly, their capacity to produce different metabolites, there are several studies that evaluate these metabolites and their applications in biotechnology. Despite these advantages, Brazil is still a long way to go to achieve what is already produced worldwide. 

There has been lot of recent research in Brazil using innovative cyanobacterial biomass from marine cyanobacteria that can generate high-value-added products, as described in this review such as dyes and food supplements complementing both animal and human food; may have anti-cancer activity and antiviral properties; and can be used as an alternative source to oil in energy production, placing Brazil in the blue biotechnology market.

Cyanobacteria and marine microalgae provide a broad field of product research and development, but they require greater focus in the form of investments in research and education, in laboratory equipment, and in training and valuing researchers, such as increasing salaries and offering training courses. 

Further studies would also be of a benefit, including work on genetic modification, as the insertion of some genes of interest, as well as the development of research involving biorefineries as Brazil is one of the largest ethanol producers in the world and also generates large volumes of residual lignocellulose, rich in sugars that can be used to nourish the cultivation of these microorganisms as the studies about changing parameters as they have already been carried out. 

The multitrophic aquaculture system is also one of the most effective ways to reduce the cost of cultivating cyanobacteria and marine microalgae, since the residual water in these systems is rich in nutrients metabolized by these microorganisms, increasing the production of PUFAs, reducing costs of nutrients, and allowing the reuse of water and the use of the biomass of cyanobacteria and microalgae as food additives for aquaculture animals. 

To make it possible to implement a microalgae industry the search for new ways to concentrate biomass and increased earnings must also be studied in order to reduce the energy, time, and expenses involved in this process so that we can reach a larger scale of production and higher yields, thus making the microalgal and marine cyanobacteria products competitive in the Brazilian market. The need to reach a global level of competitiveness is one of the concerns surrounding the booming research on marine cyanobacteria biotechnology in Brazil.

## Figures and Tables

**Table 1:** Cyanobacteria metabolites with worldwide commercial application.

Cyanobacteria	Metabolite	Application	Reference
*Spirulina platensis*	Phycocyanin	Antitumoral	[33,51]
*Nostoc commune*	MAAs	Radical Scavenging activity	[42]
*Lyngbya* sp.	Scytonemin	Photo Protection	[1]
*Synechococcus* sp.	Xanthoplyll	Photo protection	[43]
*Synechococcus* sp.	Fatty Acids	Biodiesel	[52]
Mutant *Synechococcus elongatus PCC 7942*	Omega-3	Food	[48]
*Microcystis aeruginosa*	Carbohydrate	Ethanol	[53]
*Phormidium autumnale*	Squalene	Antioxidant activity	[46]
*Sphaerospermopsis aphanizomenoides*	Ferulic Acid	Antioxidant activity	[47]
*Symploca* sp.	Largazole	Antitumoral	[36]

**Table 2:** Microalgal energy products in Brazil.

Cyanobacteria/Microalgae	Metabolite	Product	Reference
*Chlorela minutissima*	Fatty acids	Biodiesel	[57]
*Chlorella* sp.	Biomass and lipid	Biodiesel	[58]
*Isochrysis galbana* *Phaeodactylum tricornutum*	TAG	Biodiesel	[59]
*Biddulphia* sp. *Planktolynbya limnetica*	Chemical elements	Biofuels	[61]
*Synechococcus* sp.	Lipid	Biodiesel	[56]
*Spirulina* sp.	Carbohydrates	Bioethanol	[67]
*Isochrysis galbana*	Biomass	Biomethane	[71]
*Spirulina maxima*	Hydrocarbons	Bio-oil	[72]

**Table 3:** Marine species used as raw material for extraction of substances for the nutraceutical industry.

Species	Local	Application	Reference
*Tretaselmis gracilis*	Federal Fluminense University, RJ- Br/Microalgae Culture Collection.	Supplement with PUFAs	[112]
*Amphinidium carterae*	Federal University of Paraíba, PB-Br/Laboratory of Reef Envorinments and Biotechnology with Microlagae.	ω-3. (animal and human food)	[107]
*Tetraselmis gracilis*	Federal Fluminense University, RJ- Br/Microalgae Culture Collection.	Alpha-linoleic acid	[98]
*Phaeodactylumtricornutum*	Federal University of Santa Catarina, SC-BR/Algae Cultivation Laboratory	EPA	[106]
*Heterochlorella luteoviridis*	Federal Fluminense University, RJ- Br/Microalgae Culture Collection.	Carotenoid	[99]
*Phaeordactylum tricornutum*	Federal Fluminense University, RJ- Br/Microalgae Culture Collection.	Acid EPA	[113] [112]
